# 69 The Burn Is Just the Symptom: Analyzing Psychosocial Determinants of Health in the Burn Population

**DOI:** 10.1093/jbcr/irae036.061

**Published:** 2024-04-17

**Authors:** Kathryn E Harris, Patty Kalnberg, James Gallagher, Jamie M Heffernan, Anjile An, Jeremiah P Lorico, Chun Yun Hsu

**Affiliations:** New York Presbyterian/Weill Cornell Medicine, Valley Stream, New York; New York Presbyterian/Weill Cornell Medicine, New York, New York; NY Presbyterian Weill Cornell, New York, NY; New York Presbyterian/Weill Cornell, New York, New York; New York Presbyterian/Weill Cornell Medicine, Valley Stream, New York; New York Presbyterian/Weill Cornell Medicine, New York, New York; NY Presbyterian Weill Cornell, New York, NY; New York Presbyterian/Weill Cornell, New York, New York; New York Presbyterian/Weill Cornell Medicine, Valley Stream, New York; New York Presbyterian/Weill Cornell Medicine, New York, New York; NY Presbyterian Weill Cornell, New York, NY; New York Presbyterian/Weill Cornell, New York, New York; New York Presbyterian/Weill Cornell Medicine, Valley Stream, New York; New York Presbyterian/Weill Cornell Medicine, New York, New York; NY Presbyterian Weill Cornell, New York, NY; New York Presbyterian/Weill Cornell, New York, New York; New York Presbyterian/Weill Cornell Medicine, Valley Stream, New York; New York Presbyterian/Weill Cornell Medicine, New York, New York; NY Presbyterian Weill Cornell, New York, NY; New York Presbyterian/Weill Cornell, New York, New York; New York Presbyterian/Weill Cornell Medicine, Valley Stream, New York; New York Presbyterian/Weill Cornell Medicine, New York, New York; NY Presbyterian Weill Cornell, New York, NY; New York Presbyterian/Weill Cornell, New York, New York; New York Presbyterian/Weill Cornell Medicine, Valley Stream, New York; New York Presbyterian/Weill Cornell Medicine, New York, New York; NY Presbyterian Weill Cornell, New York, NY; New York Presbyterian/Weill Cornell, New York, New York

## Abstract

**Introduction:**

There is an understanding in the burn community that burns occur as a result of something, whether it be medical conditions, psychiatric illness, poverty, living conditions, etc. Social determinants of health (SDOH) are defined as non-medical factors that can affect one’s health. These factors, along with psychiatric illness, can impact inpatient care and discharge planning. Our objective was to determine the prevalence of psychosocial factors in our burn population and analyze their impact on length of stay (LOS) and discharge disposition.

**Methods:**

A retrospective chart review from January 1, 2022 to December 31, 2022 was performed at a large, urban, American Burn Association (ABA)-verified burn center using data from the ABA Burn Care Quality Platform (BCQP). All admitted patients ≥18 years old who sustained a burn injury were included. The two SDOH captured by BCQP, insurance status and living situation, were collected, in addition to psychiatric diagnosis, substance use disorder and alcohol use disorder. Primary outcome was LOS and secondary outcome was discharge disposition. Continuous variables were represented as median (interquartile range) and categorical variables as n (%). The Wilcoxon rank-sum test and Fisher’s exact test were used to compare LOS and discharge disposition between psychosocial variables of interest.

**Results:**

Out of 302 patients, 129 (43%) had at least one negative SDOH. Of these 129 patients, 97% of them were uninsured/underinsured and 14% were undomiciled. Of the total population, 16% had a psychiatric diagnosis, 4.6% had alcohol use disorder and 8.6% had substance use disorder with a higher prevalence in the uninsured/underinsured and undomiciled population. There were 18 patients (6%) who were undomiciled prior to admission. The median LOS for all 302 patients was 8 days (IQR: 2, 17) and was not significantly different by insurance. Patients with a psychiatric diagnosis had significantly longer median LOS (15 days) when compared to those without (7 days, p=0.009). Undomiciled patients also had significantly longer median LOS (12 days vs 7 days, p = 0.034). Comparing by insurance, patients were discharged home with self-care at a similar rate (68% for insured, 62% for uninsured/underinsured). Patients in the uninsured/underinsured group were less likely to go to subacute rehab (4.3%) compared to the well-insured (11%). Only 28% of undomiciled patients were discharged back to a shelter or street.

**Conclusions:**

The care of our burn patients is not defined by insurance status. However, the burn community needs a better, systematic process of collecting additional SDOH to further understand the impact they have on our patients. The longer LOS for those with psychiatric illness and homelessness highlights the need for specialized care both in the inpatient and post-discharge setting.

**Applicability of Research to Practice:**

Directly applicable

